# Parasitic Current Induced by Gate Overlap in Thin-Film Transistors

**DOI:** 10.3390/ma14092299

**Published:** 2021-04-29

**Authors:** Hyeon-Jun Lee, Katsumi Abe, June-Seo Kim, Won Seok Yun, Myoung-Jae Lee

**Affiliations:** 1 Institute of Convergence, Daegu Gyeonbuk Institute of Science & Technology (DGIST), Daegu 42988, Korea; spin2mtj@dgist.ac.kr (J.-S.K.); wsyun@dgist.ac.kr (W.S.Y.); myoungjae.lee@dgist.ac.kr (M.-J.L.); 2 Silvaco Japan Co., Ltd., Nakagyo-ku, Kyoto 604-8152, Japan; katsumi.abe@silvaco.com

**Keywords:** oxide semiconductor, *a*-IGZO, barrier lowering, hump

## Abstract

As novel applications of oxide semiconductors are realized, various structural devices and integrated circuits are being proposed, and the gate-overlay defect phenomenon is becoming more diverse in its effects. Herein, the electrical properties of the transistor that depend on the geometry between the gate and the semiconductor layer are analyzed, and the specific phenomena associated with the degree of overlap are reproduced. In the semiconductor layer, where the gate electrode is not overlapped, it is experimentally shown that a dual current is generated, and the results of 3D simulations confirm that the magnitude of the current increases as the parasitic current moves away from the gate electrode. The generation and path of the parasitic current are then represented visually through laser-enhanced 2D transport measurements; consequently, the flow of the dual current in the transistor is verified to be induced by the electrical potential imbalance in the semiconductor active layer, where the gate electrodes do not overlap.

## 1. Introduction

Since Nomura et al. [1] presented metal–oxide–semiconductor thin-film transistors as replacements for silicon-based devices in active matrix displays, numerous studies have been intensively conducted on this topic for over a decade [2,3,4,5,6,7,8]. These studies have primarily focused on emerging applications [9] as well as defects [10,11,12]. As the complexity of the semiconductor device structure increases and the gate, active, and source/drain (S/D) lengths of the physical dimension decrease, the likelihood of a defect occurring where the gate overlaps with the active layer increases. This can reduce the device’s lifetime or lead to a completely defective device due to unexpected electrical properties.

The overlap between the active layer (semiconductor), located between the source and drain, and gate electrodes is a very important factor in determining the characteristics of the semiconductor. The electrical properties of semiconductors affect the on/off state of the device by changing the gate voltage. The overlap of the gate electrode and S/D has a significant influence on the capability of circuit configuration, which then significantly impacts the overall running speed of the circuit [13]. This overlap can generate parasitic currents (“hump”), which, in turn, change the electrical properties [14,15,16]. Previous studies have been limited in scope and have investigated individual effects such as the vertical overlap of gate electrodes and S/Ds [13,17] or the parasitic current known as the “hump effect” [15]. In particular, there have been many reports on the parasitic current that is observed in characteristic anomalies due to defect generation, occurring during reliability tests [18,19,20,21,22,23,24,25] or semiconductor fabrication processes [26]. However, there are no studies on the lateral geometry between the active layer and the gate electrode, which is an inevitable defect phenomenon in high-density integrated devices.

In this work, we investigate the effect of electrical characteristics due to the lateral active-gate overlap alignment (the half-gate structure) of transistors that can be caused by high-density integrated circuits or device processes. The electrical characteristics of the transistor associated with the overlap of the gate electrode to the active layer were experimentally measured, and abnormal electrical characteristics were identified through numerical computational simulations. In particular, using laser-enhanced 2D transport measurements, the existence and path of the parasitic current were verified by visualizing the path where the parasitic current is generated.

## 2. Devices and Experiment

**Figure 1:**
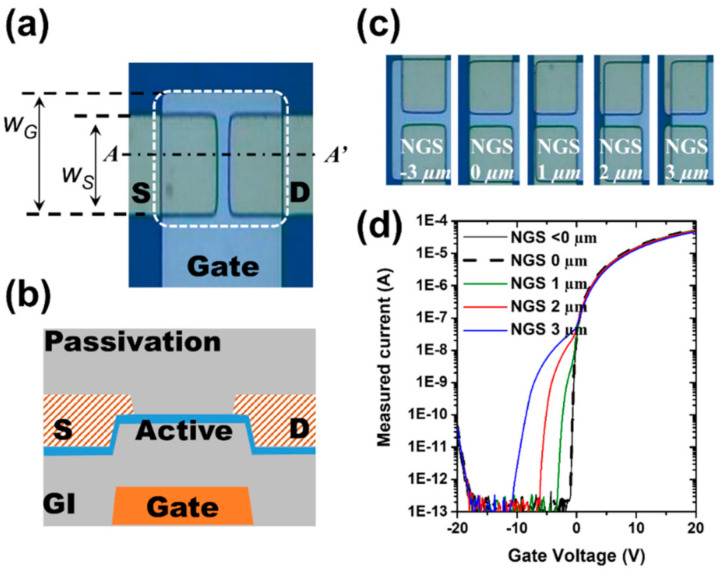
(**a**) Top view of TFT sample, (**b**) cross-sectional schematic image at *A-A’* cut-line, (**c**) five different samples as a function of NGS values, and (**d**) I_d_-V_g_ transfer characteristics depending on NGS values.

An amorphous InGaZnO_x_ (*a*-IGZO) thin film transistor (TFT) with an inverted-stage structure was used in this study, as shown in Figure 1a,b. Mo gate electrodes (100 nm) were formed on an amorphous SiO_x_/Si substrate, and 150-nm-thick SiN_x_/50 nm SiO_x_ dual layers were deposited as the gate insulator (GI) at 350 °C by chemical vapor deposition (CVD). An *a*-IGZO layer with a thickness of 40 nm was deposited at 100 °C on the SiO_x_ GI by RF magnetron sputtering. The Mo 100 nm source and drain electrodes were formed via photolithography. Dual passivation layers of SiO_x_ 50 nm/SiN_x_ 150 nm were prepared by CVD on the active layer at 350 °C. The *a*-IGZO TFT was annealed in ambient air at 350 °C for 1 h, and thereafter, the contact holes were defined by photolithography and reactive ion etching processes. Five different samples (Figure 1c) were fabricated with variations in the overlap of the gate electrode and active area. The active region exists under the S/D region (the channel width/length is 17/4 and the width of S/D is 17.5 μm), and it was difficult to distinguish the active region in the picture because we used transparent IGZO as the active layer semiconductor. The region of non-overlap between the gate and source edges (NGS) is generally less than zero based on the definition in Equation (1), and distortion of the electric field may occur, resulting in abnormal electrical characteristics.

(1) $$\mathrm{NGS}=W_{S}-W_{G}$$

The NGS represents the difference between the size of the gate (*W_G_*) and the size of the source electrode (*W_S_*) relative to the edge below the source electrode, as depicted in Figure 1a. Current vs. voltage (I–V) measurement (gate voltage sweeps from −20 V to 20 V) was carried out under V_ds_ = 10 V using the Keithley 2636B (Keithley, Solon, OH, USA).

## 3. Results and Discussion

As shown in Figure 1d, electrical characteristics were evaluated for five types of devices, each with a fixed channel length of 4 μm and NGS values ranging from −3 to 3 μm. The electrical properties of the samples with negative and 0 μm NGS values were the same, and these are indicated by dashed lines. As NGS values increased from 1 to 3, it was confirmed that the current in the negative gate voltage region increased. Moreover, the hump phenomenon was induced by the NGS structure. The mobility, the threshold voltage, and the sub-threshold swing of the normal TFT (NGS = 0 μm) are confirmed to be 6.4 cm2/Vs, −0.5 V, and 190 mV/dec, respectively.

**Figure 2:**
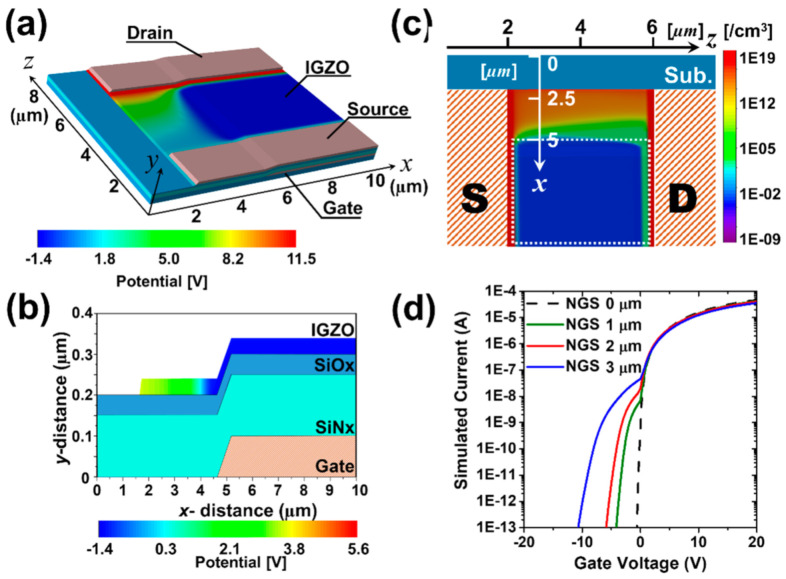
(**a**) Three-dimensional potential distribution at NGS of 3 µm, (**b**) the potential distribution in the *x-y* cut-plane at *z* = 4 µm, (**c**) electron concentration of the top view image (the white dashed-box indicates the gate electrode position under the active layer), and (**d**) the simulated transfer characteristics as a function of the NGS values.

Three-dimensional device TCAD (Technology computer-aided design) simulations (Atlas Device simulator, Silvaco Inc., Santa Clara, CA, USA) were performed to confirm the correlation between the NGS structure and the occurrence of the hump phenomenon. Since abnormal characteristics are shown by the overlap of the gate, the simulation was conducted around the area where the gate overlap occurs. The device used in the simulation was 8 µm (*z* axis) × 10 µm (*x* axis) in size, and the channel length was fixed at 4 µm (*z* axis) (Figure 2a). Four types of unit devices (NGS values of 0, 1, 2, and 3 µm) were prepared to evaluate the electrical properties experimentally. When 10 V was applied between the source and the drain at V_g_ = −7.5 V, the potential distribution in the NGS 3 µm structure was calculated in the 3D simulations, as shown in Figure 2a. Although a negative voltage of −7.5 V is applied to the gate electrode, a relatively high potential distribution appears in the NGS region. To confirm the potential distribution based on the *x*-axis, the *x–y* cut-plane analysis at *z* = 4 µm was performed, as shown in Figure 2b. The potential in the *x* = 5–10 µm region under the influence of the gate electrode has a negative value, while the NGS region, which is not directly affected by the gate electrode, has a relatively high potential. In particular, Figure 2b shows that the potential near *x* = 2 µm, the area farthest from the gate electrode, is strongly generated. Owing to the influence of the potential distribution, an imbalance of electron concentration was observed in the NGS region and the active region above the gate. Figure 2c is a top view image displayed on a logarithmic scale of the electron concentration distribution of a 3D structure. It was observed that the distribution of electron concentration changes exponentially near the boundary at approximately *x* = 5 µm. This section may be divided by subheadings. It should provide a concise and precise description of the experimental results, their interpretation, as well as the experimental conclusions that can be drawn.

**Figure 3:**
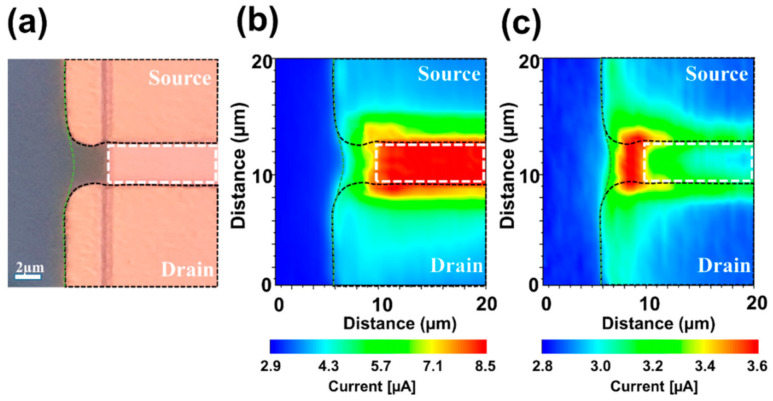
(**a**) High-resolution microscopy image of the device with an NGS of 3 µm, and the photocurrent signal at (**b**) V_g_ = 20 V and (**c**) V_g_ = −7.5 V. The white and green dashed lines indicate the gate electrode and the boundary of the active layer, respectively. The black dashed line is the guide for the S/D electrode.

It was confirmed that the hump effect can occur due to the creation of the NGS structure, and the flow of this parasitic current is due to the potential asymmetry imbalance in the NGS region. In addition, TCAD simulations showed that most of the current flows through the NGS region when the gate is OFF. To experimentally confirm the current flow to the NGS region observed in the simulation results, laser-enhanced 2D transport measurements were conducted [27]. By using a sample with an NGS of 3 µm, 2D transport measurements were performed for the ON (V_g_ = 20 V) and OFF (V_g_ = −7.5 V) states of the gate electrode at V_ds_ = 10 V. Prior to proceeding with the 2D transport evaluation, high-resolution microscopy images were obtained for the evaluation area (Figure 3a). A 405-nm laser was used to scan ±10 µm around the NGS structure, and the photocurrent generated at each location was measured. Figure 3b shows the measured photocurrent by position when the gate voltage is ON (V_g_ = 20 V). It can be seen that a strong photocurrent is observed only in the gate area, as indicated by the white dashed line. In general, the injection of a 405-nm laser generates equal photocurrents at all positions in *a*-IGZO. However, as shown in Figure 3b, when laser is injected at V_g_ = 20 V/V_ds_ = 10 V, photocurrent is observed only in the area directly affected by the gate electrode. In contrast, when the gate is OFF, a large amount of photocurrent is observed in the NGS area, as shown in Figure 3c, but there is no signal at the top of the gate (the white dashed box).

**Figure 4:**
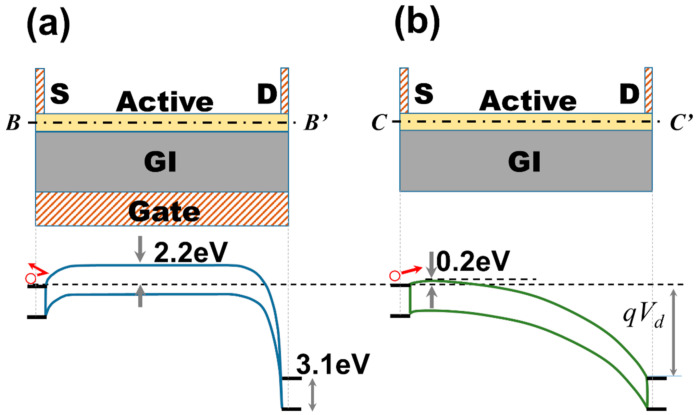
Energy band diagram for *y-z* cut-plane; (**a**) at *x* = 8 µm (with the gate electrode), and (**b**) at *x* = 2.3 µm (without the gate electrode) for V_ds_ = 10 V and V_g_ = −7.5 V.

The electrical characteristics may vary depending on the overlap between the gate electrode and the S/D, and the overlap between the gate electrode and the active area. In particular, it was confirmed that hump characteristics can be induced in the NGS structure. In addition, it was experimentally confirmed that this phenomenon occurs owing to the flow of current via the NGS region when the gate is in the OFF state. This phenomenon could be understood as the energy band diagram (calculated during device simulation) shown in Figure 4. In general, when the gate is OFF, electrons cannot enter the active layer from the source electrode, as shown in Figure 4a. This is due to the potential barrier near the source electrode formed in the active layer. However, in the NGS area without the gate electrode (positioned at *x* = 2.3 µm), it can be seen that the potential barrier near the source electrode is very low. This is similar to drain-induced barrier lowering (DIBL) where a large leakage current can flow between the source and drain, as the drain bias can affect the barrier at the end of the source when the source and drain are very close [28]. However, in this study, as the S/D was not sufficiently close (the distance between S/D is 4 µm), the drain did not affect the source barrier. Specifically, in this study, the effect of lowering the potential barrier near the source was caused by the imbalanced potential generated in the active layer in the NGS region, which can cause parasitic current.

## 4. Conclusions

In this study, we report on the abnormal electrical properties resulting from the overlap of gate electrodes and active layers in a half-gate thin-film transistor structure. Although the electric potential is weakened in areas where the gate is not overlapped by the active layer, an insulation layer is created inside the active zone by the imbalanced potential. The generation of these depletion regions distorts the flow of electrons. This method is expected to be useful in the simulated analysis of the electrical characteristics and defects of gate electrodes for improving their application and fabrication.

## Data Availability

The data presented in this study are available on request from the corresponding author.

## References

[1] Nomura K., Ohta H., Ueda K., Kamiya T., Hirano M., Hosono H. (2003). Thin-film transistor fabricated in single-crystalline transparent oxide semiconductor. Science.

[2] Takahashi T., Fujii M.N., Miyanaga R., Miyanaga M., Ishikawa Y., Uraoka Y. (2020). Unique degradation under AC stress in high-mobility amorphous In–W–Zn–O thin-film transistors. Appl. Phys. Express.

[3] Lee H., Abe K. (2020). A Study on the Effect of Pulse Rising and Falling Time on Amorphous Oxide Semiconductor Transistors in Driver Circuits. IEEE Electron Device Lett..

[4] Takahashi T., Miyanaga R., Fujii M.N., Tanaka J., Takechi K., Tanabe H., Bermundo J.P., Ishikawa Y., Uraoka Y. (2019). Hot carrier effects in InGaZnO thin-film transistor. Appl. Phys. Express.

[5] Noh H.Y., Kim J., Kim J.-S., Lee M.-J., Lee H.-J. (2019). Role of Hydrogen in Active Layer of Oxide-Semiconductor-Based Thin Film Transistors. Crystals.

[6] Hong S.Y., Kim H.J., Kim D.H., Jeong H.Y., Song S.H., Cho I.T., Noh J., Yun P.S., Lee S.W., Park K.S. (2019). Study on the Lateral Carrier Diffusion and Source-Drain Series Resistance in Self-Aligned Top-Gate Coplanar InGaZnO Thin-Film Transistors. Sci. Rep..

[7] Yang C., Chang T., Liao P., Chen L., Chen B., Chou W., Chen G., Lin S., Yeh C., Tsai C. (2018). Drain-Induced-Barrier-Lowing-Like Effect Induced by Oxygen-Vacancy in Scaling-Down via-Contact Type Amorphous InGaZnO Thin-Film Transistors. IEEE J. Electron Devices Soc..

[8] Myny K. (2018). The development of flexible integrated circuits based on thin-film transistors. Nat. Electron..

[9] Chen H.T., Cao Y., Zhang J.L., Zhou C.W. (2014). Large-scale complementary macroelectronics using hybrid integration of carbon nanotubes and IGZO thin-film transistors. Nat. Commun..

[10] Ide K., Nomura K., Hosono H., Kamiya T. (2019). Electronic Defects in Amorphous Oxide Semiconductors: A Review. Phys. Status Solidi.

[11] Fortunato E., Barquinha P., Martins R. (2012). Oxide semiconductor thin-film transistors: A review of recent advances. Adv. Mater..

[12] Lee H.-J., Cho S.H., Abe K., Lee M.-J., Jung M. (2017). Impact of transient currents caused by alternating drain stress in oxide semiconductors. Sci. Rep..

[13] Lee S., Li X., Mativenga M., Jang J. (2015). Bulk-Accumulation Oxide Thin-Film Transistor Circuits with Zero Gate-to-Drain Overlap Capacitance for High Speed. IEEE Electron Device Lett..

[14] Jeong J., Hong Y. (2010). Gate Overlap Optimization and Performance Variation for Thin-Film Transistors with Source/Drain Edge Waviness. Jpn. J. Appl. Phys..

[15] Lee H.-J., Abe K., Noh H.Y., Kim J.-S., Lee H., Lee M.-J. (2019). Analysis of the hump phenomenon and needle defect states formed by driving stress in the oxide semiconductor. Sci. Rep..

[16] Mativenga M., Haque F., Um J.G., Siddik A.B. (2020). Impact of Source-to-Gate and Drain-to-Gate Overlap Lengths on Performance of Inverted Staggered a-IGZO TFTs with an Etch Stopper. IEEE Trans. Electron Devices.

[17] Valletta A., Gaucci P., Mariucci L., Fortunato G., Templier F. (2008). “Hump” characteristics and edge effects in polysilicon thin film transistors. J. Appl. Phys..

[18] Huang C.F., Peng C.Y., Yang Y.J., Sun H.C., Chang H.C., Kuo P.S., Chang H.L., Liu C.Z., Liu C.W. (2008). Stress-Induced Hump Effects of p-Channel Polycrystalline Silicon Thin-Film Transistors. IEEE Electron Device Lett..

[19] Mativenga M., Seok M., Jang J. (2011). Gate bias-stress induced hump-effect in transfer characteristics of amorphous-indium-galium-zinc-oxide thin-fim transistors with various channel widths. Appl. Phys. Lett..

[20] Choi S.-H., Han M.-K. (2012). Effect of channel widths on negative shift of threshold voltage, including stress-induced hump phenomenon in InGaZnO thin-film transistors under high-gate and drain bias stress. Appl. Phys. Lett..

[21] Tsai M.-Y., Chang T.-C., Chu A.-K., Hsieh T.-Y., Chen T.-C., Lin K.-Y., Tsai W.-W., Chiang W.-J., Yan J.-Y. (2013). High temperature-induced abnormal suppression of sub-threshold swing and on-current degradations under hot-carrier stress in a-InGaZnO thin film transistors. Appl. Phys. Lett..

[22] Jeong C.-Y., Lee D., Song S.-H., Kim J.I., Lee J.-H., Kwon H.-I. (2014). A study on the degradation mechanism of InGaZnO thin-film transistors under simultaneous gate and drain bias stresses based on the electronic trap characterization. Semicond. Sci. Technol..

[23] Hwarim I., Hyunsoo S., Jaewook J., Yewon H., Yongtaek H. (2015). Effects of defect creation on bidirectional behavior with hump characteristics of InGaZnO TFTs under bias and thermal stress. Jpn. J. Appl. Phys..

[24] Lee H.-J., Abe K., Kim J.S., Lee M.-J. (2017). Electron-blocking by the potential barrier originated from the asymmetrical local density of state in the oxide semiconductor. Sci. Rep..

[25] Lee H.-J., Abe K., Cho S.H., Kim J., Bang S., Lee M. (2018). Drain-Induced Barrier Lowering in Oxide Semiconductor Thin-Film Transistors with Asymmetrical Local Density of States. IEEE J. Electron Devices Soc..

[26] Furuta M., Kamada Y., Kimura M., Hiramatsu T., Matsuda T., Furuta H., Li C., Fujita S., Hirao T. (2010). Analysis of Hump Characteristics in Thin-Film Transistors with ZnO Channels Deposited by Sputtering at Various Oxygen Partial Pressures. IEEE Electron Device Lett..

[27] Lee M.-J., Ahn J.-H., Sung J.H., Heo H., Jeon S.G., Lee W., Song J.Y., Hong K.-H., Choi B., Lee S.-H. (2016). Thermoelectric materials by using two-dimensional materials with negative correlation between electrical and thermal conductivity. Nat. Commun..

[28] Sze S.M., Li Y., Ng K.K. (2006). Physics of Semiconductor Devices.

